# The Case for Protective Headguards in Amateur Boxing and Implications for International Policy on Headguard Bans

**DOI:** 10.1186/s40798-025-00871-4

**Published:** 2025-05-16

**Authors:** Kelvin Le, James Norton Marchant, Khang Duy Ricky Le

**Affiliations:** 1 Melbourne Medical School, The University of Melbourne, Melbourne, VIC 3000 Australia; 2 Department of General Surgical Specialties, The Royal Melbourne Hospital, Melbourne, VIC 3052 Australia; 3 Deakin School of Medicine, Faculty of Health, Deakin University, Geelong, VIC 3220 Australia; 4 Department of Medical Education, Melbourne Medical School, The University of Melbourne, Melbourne, VIC 3000 Australia

**Keywords:** Boxing, Headguards, Cuts and Lacerations, Concussion, Protection, Safety, Policy

## Abstract

The recent prohibition of headguards by the International Boxing Association (IBA; previously AIBA) has sparked worldwide controversy regarding safeguarding the health of athletes in the boxing community. Studies have evaluated the role of headguards in preventing concussion, cuts and lacerations and fractures. However, the evidence for improvement in these outcomes remains poorly characterised and a gap remains in the ability to inform evidence-based sport and health policy in this space. This Current Opinion article demonstrates the effectiveness of headguards in protecting against cuts and lacerations and notes there is insufficient evidence supporting headguard bans. Furthermore, this article highlights a gap in collaborative effort and communication between the IBA and smaller representative bodies, which is an important consideration for future policy reform.

## Key Points

Headguards in amateur boxing are effective in protecting against facial cuts and lacerations.

There is a need for more robust research as a whole in characterising the efficacy of headguards in preventing concussion and craniofacial injuries in amateur boxing.

## Introduction

Amateur boxing carries the inherent risk of head injury, ranging from superficial cuts and concussions to severe intracranial trauma [1, 2]. The priorities for sporting bodies therefore have been on athlete safety, particularly through refining regulations in amateur boxing. Among this is policy on headguard use. At the 1984 Los Angeles Olympic Games, headguards were introduced to prevent superficial injuries in boxing. Decisions surrounding this headguard mandate were implemented in response to banning threats made by the American Medical Association in the context of safety concerns for amateur boxing athletes [1, 3, 4]. Headguards were also intended to limit the number of direct blows and injuries to the head, which were causing bouts to be rendered ‘won’ prematurely with early stoppage times [2, 5]. Furthermore, it is important to note that at the time of this decision, there was no evidence demonstrating the effectiveness of headguards in reducing concussion rates [6].

In 2013, the International Boxing Association (IBA; formally AIBA) made the controversial decision to ban headguards in male senior open-class boxing competitions [7]. This decision was predicated on the belief that headguards offered a false sense of security, potentially encouraging riskier behaviour and not providing adequate protection against concussions [1, 2]. Additionally, the reasoning was seen as secondary to an attempt to satisfy the media and spectator community, by taking the game'back to its roots' [4]. Significant opposition has emerged against the IBA rule change, underscoring a critical concern over what many perceive as a disregard for athlete safety [7].

In spite of this, in a move that seeks to align female boxers with their male counterparts, the IBA has extended this rule to women [8]. This decision reignites discourse on the necessity and efficacy of protective gear in amateur boxing. Importantly, this decision also raises important questions regarding fostering a culture that promotes the valorisation of dominance and aggression, or one that prioritises the safety of its athletes. This article examines the rationale behind this policy change from an evidence-based perspective through first examining the impacts of headguards on common boxing injuries. The article then provides evidence-informed insights into the necessary considerations for effective headguard policy reform in amateur boxing with athlete safety at the forefront.

## The Role of Headguards in Preventing Injuries in Boxing

### Concussion

The prevention of concussions has been an indication for implementation of headguards in amateur boxing. Boxers’ susceptibility to brain injuries have been well recorded, with a study demonstrating that up to 71% of injuries were localised to the head region (athlete exposure = 10721.1 h in a 12 month period) [9]. Such impact to the head can lead to significant acceleration-deceleration injuries that cause neuronal and vascular damage [10]. Among head injuries, concussions are the most prevalent, accounting for 33% of cases [9]. However there is significant heterogeneity in concussion rates reported in the literature, ranging from 6.1 to 75% [2, 11]. This variability appears to stem from two factors. The first concerns how concussions are defined and diagnosed on the background of a poor quality evidence base with high heterogeneity [7]. Secondly, the existing literature examining the incidence of concussions before and after the IBA headguard ban does not adequately collect data on this metric. Instead, it uses the frequency of punches to the head and stoppages from head strike as surrogate measures, leading to inconsistencies in interpretation [3, 12–14].

Studies that have evaluated headguards note their effectiveness in reducing peak impact force, as well as linear and angular head accelerations [15, 16]. However, there is a paucity of evidence demonstrating their role in mitigating concussions or traumatic brain injuries [2]. Additionally, the fact that the headguard rule was introduced alongside a suite of new rules introduces additional confounding variables in the assessment of headguard efficacy.

### Cuts and Lacerations

Facial cuts and lacerations are estimated to account for 29% of reported injuries (athlete exposure = 10721.1 h in a 12 month period) [9, 16]. Some studies however suggest these injuries represent the most prevalent craniofacial injury in boxing [9, 17]. The intent to implement headguards therefore revolves around reducing the incidence of these injuries [1, 4]. Furthermore, in addition to safety, these injuries are also relevant as they can lead to athlete disqualification from further competition, thus having both financial and medical consequences [4, 7]. There is a noticeable lack of evidence surrounding whether headguards improve the rates of cuts and lacerations. To date, there are currently two studies that have investigated the effects of headguards for this purpose. Loosemore et al. [13] performed a prospective cross observational study investigating the incidence of these injuries before and after the 2013 IBA headguard ban. They found that following headguard bans, rates of facial cuts and lacerations increased by 430% (RR = 5.30; number of cuts or lacerations with headguard use = 45, 60.5/1000 h; without headguard use = 223, 320.4/1000 h) over a 4-year period. Additionally, the authors conducted a case study comparison of the IBA World Championships in 2009 (headguard use), 2011 (headguard use) and 2013 (no headguard use). This similarly highlighted a greater incidence of facial cuts and lacerations following headguard removals (number of cuts or lacerations– 2009: 36.7/1000 h, 2011: 35.71/1000 h, 2013: 320.65/1000 h) [13]. Bianco et al. [12] performed a retrospective web-based analysis of stoppages related to referee-stop contest (RSC) decisions dictated by injuries including cuts and fractures (RSCI) between 1952 and 2011. The authors found a significant reduction of RSCI decisions by 3.3-fold after the introduction of headguards in 1984 (*P* < 0.001). Although not entirely focused on cuts and lacerations, Bianco et al. [12] demonstrate evidence further suggesting reduced incidences of cuts and lacerations with headguard use [12]. Overall, although limited by few studies with small sample sizes, the current evidence suggests the effectiveness of headguards in reducing facial cuts and lacerations.

### Skull Fractures

Skull fractures are a less common injury, but confer greater morbidity. Observational studies suggest they comprise 19% of reported injuries (athlete exposure = 10721.1 h in a 12 month period) [9]. Many studies have commented on the effectiveness of headguards in preventing skull fractures in boxing [2, 17]. However, there are limited studies explicitly exploring the influence of headguards on skull fracture rates and their downstream sequelae in amateur boxing. Bianco et al.. demonstrated a 3.3-fold reduction in RSCI (*P* < 0.001) after the introduction of headguards in 1984 [12]. This suggests that headguards have a role in reducing fracture rates, although the quality of this evidence is poor. The influence of headgear on skull fractures therefore remains poorly characterised and further rigorous research is required to evaluate this association.

### Nasal Injuries

Nasal injuries are another less common injury, representing 19% of reported injuries (athlete exposure = 10721.1 h in a 12 month period) [9]. A recent study by Al-Awady et al. [18] comparing emergency department presentations of nasal injuries before and after the 2013 headguard bans between 2006 and 2021found significant reductions following headguard removal in total injuries (6261 vs. 5499, *P* < 0.001), contusions (1133 vs. 982, *P* = 0.019), fractures (4140 vs. 3666, *P* = 0.004), haematomas (102 vs. 5, *P* < 0.001) and haemorrhage (446 vs. 306, *P* < 0.001). Contrarily, there were significant increases in non-specific nasal injury (187 vs. 267, *P* < 0.001) and septal deviation (16 vs. 97, *P* < 0.001). Finally there were no significant changes in nasal lacerations (225 vs. 188, *P* = 0.186). Overall, this study largely demonstrates increased incidences of nasal injuries associated with headguard use [18].

## Psychological and Behavioural Factors Surrounding Headguard Use

Anecdotally, headguards confer a sense of safety by providing an additional layer of protection against craniofacial injuries. Curiously, there is a suggestion that this is associated with reduced defensive strategies and increased risky behaviours in boxing; dubbed “leading with the head” [4]. These behavioural factors form the basis of recent IBA headguard bans and educational initiatives such as ‘#HeadsUp!’, established to promote strategies focusing on preventing head injuries [19, 20]. Behavioural changes before and after the 2013 headguard bans in boxing have been analysed by two studies. Davis et al. [3, 21] found drastic changes in boxing strategies after retrospective observation of bouts prior to and after the 2013 headguard bans. They suggest strategies post-2013 prioritised avoidance of head injuries and focused more on defensive maneuvers (*P* = 0.004) including increased single punches thrown (in comparison to combinations) (*P* = 0.023) and decreased punches with the rear hand (*P* = 0.023). Additionally, longer range strategies were implemented after headguard removal, as indicated by increased frequency of straight blows (*P* = 0.005) and decreased frequency of hooks (*P* = 0.042). Overall, the authors commented that the styles of boxing involved greater foot movement and ‘in and out’ defensive style. However, this study also found an increased number of head blows (*P* < 0.001) and decreased number of body blows (*P* < 0.001), which suggests the removal of headguards may promote the head as a primary target [3, 21]. Moreover, Loosemore et al. [13] demonstrated a near-half reduction of stoppages from head blows in bouts without headguards (*n* = 23; 33/1000 h) compared to with headguards (*n* = 43; 57.8/1000 h). Additionally, there were fewer stoppages from head blows in the World Championships without headguard use compared to with headguard use (number of stoppages from head blows– 2009: 42.81/1000 h, 2011: 41.67/1000 h, 2013: 14.91/1000 h) [13]. On a similar note, Bianco et al. found higher rates of RSC stoppages from blows to the head (RSCH) following the introduction of headguards in 1984 (*P* < 0.001) [12]. Altogether, these studies highlight evidence of the psychological safety that is associated with headguard use and consequential adaptation into more defensive and avoidance-based strategies following headguard removal. Whether this has influence on the effectiveness, redundancy, or even potential harm of headguard use however remains unclear.

## The Influence of Policy on Headguards in Amateur Boxing and Areas for Reform

Current policies governing the use or removal of headguards are sanctioned by the IBA. These policy reforms were based on internal, unpublished studies conducted by the IBA Medical Commission that analysed ‘more than 2000 bouts’, with the intent of protecting athlete safety [7, 20]. To support the removal of headguards, the IBA established educational initiatives including #HeadsUp! to promote strategies involving avoidance of head injuries to athletes and their coaching teams, but the impacts of these advocacy campaigns are poorly understood [19, 20]. From our review of the literature, studies have shown variable evidence surrounding the efficacy of headguard bans, acknowledging a key contributor to this being heterogeneity of the evidence and lack of longitudinal follow-up data assessing long term outcomes due to repetitive trauma, such as in chronic traumatic encephalopathy [22]. Therefore, the decision to remove headguards in amateur boxing remains controversial, with evidence supporting headguard use for protection against facial cuts, lacerations and skull fractures but contradictory evidence for nasal injuries [1, 12, 18]. Furthermore, the arguments about whether the improved outcomes in terms of reducing concussion and other traumatic brain injury related events are confounded by the effects of additional rules implemented concomitantly with the headguard bans [2, 12]. Despite this, the IBA continues to endorse the removal of headguards in boxing, with recent advancements made in banning headguard use in women’s amateur boxing as of March 2024 [8].

National boxing bodies and medical organisations have continued to express disapproval with the IBA decision to remove headguards. Within the World Medical Association Statement on Boxing, Sect. 7.5 continues to recommend ‘head gear’ as a personal protective equipment [23]. Furthermore, one of the most well-documented advocacy efforts against headguard bans involves the Canadian boxing community. Dickinson and Rempel conducted an online poll-based study exploring the opinions on headguard use from members of the Canadian boxing community [7]. The consensus was large-scale disapproval of the bans, with 71.5% of respondents (*n* = 431) opposed to any removal of headguards, whilst 5.8% of respondents endorsed their prohibition [7]. From the viewpoint of active boxers (*n* = 154), 58.4% opposed any form of headguard removal, 35.7% were in favour of conditional headguard use, and 5.8% endorsed their prohibition [7]. Overall, the authors demonstrated an overall disapproval of IBA decision to remove headguard use in amateur boxing [7]. Since this study, recent developments in Canada include the formal decision to reinstate headguard use in amateur Canadian boxing bouts, as mandated by Boxing Canada since 2019 [24]. This decision was largely in response to greater rates of cuts and lacerations observed following the headguard bans, a preventable injury which has hindered many athletes from progressing in competitions. Furthermore, the President of Boxing Canada, Pat Fiacco, commented on the lack of evidence in reduction of concussion rates since the IBA decision to remove headguards, which was the primary justification for their initial removal [24]. Other national organisations that have similarly reinstated mandatory headguard use include USA Boxing and the Argentina Boxing Federation. Although it appears that headguard policies can be influenced at a national level, it is important to appreciate the overarching influence that IBA policy holds in international settings. That is, athletes must continue to comply with the IBA rules in international competitions, which include headguard bans [24].

Finally, the ethical and moral effects of headguard policies should be acknowledged. It is important to consider whether there are underlying factors outside of health that may influence the headguard bans. Boxing without headguards may be more attractive entertainment-wise and appeal to mass audiences [4, 12]. Furthermore, these policies may elicit moral predicaments at an individual level, such as whether athletes are comfortable in participating without headguards [7]. This raises dilemmas that policy makers must face with respect to undue influence on athlete autonomy, particularly in a high-risk injury prone sport like boxing. Moreover, at the time of the initial ban, there were unaligned expectations between IBA and other boxing associations. In Australia, the decision to remove headguards conflicted with Boxing Australia insurance policies, which explicitly did not cover athletes without headguards. Ramifications include participating in bouts that were not officially recognised if headguards were used, or boxing uninsured, with boxers having reported doing ‘whatever it takes’ to ‘get in the ring’ [25]. This highlights potential gaps in communication and mutualistic collaboration with smaller bodies that the IBA represent in policy making regarding athlete safety [7]. These lessons provide impetus for policy makers and relevant stakeholders to consider that promoting athlete safety requires both headguard policy reform that prioritises the wellbeing of athletes in addition to fostering greater collaboration with smaller amateur boxing bodies to ensure that mutual expectations, consensus and inputs are respected.

## Conclusion

With controversy surrounding the recent headguard bans in amateur boxing, studies have attempted to elucidate the effectiveness of headguards primarily through incidences of craniofacial injuries, with particular emphasis on concussions, cuts and lacerations, and fractures. Justifications for the IBA ban on headguards are markedly weak against the backdrop of studies underscoring their protective effects, specifically in preventing cuts and lacerations. Future policy reform should be informed by a collaborative approach with smaller amateur boxing bodies to ensure a comprehensive and safety-oriented framework for the sport. This stance aligns with the overarching goal of prioritising athletes’ well-being while maintaining the integrity and appeal of amateur boxing.

## Data Availability

All data discussed and analysed in this article are from publicly available peer reviewed articles obtained from the medical database Medline. Data drawn from the included articles were in their original form and not modified. Data were referenced accordingly in the Reference list to acknowledge their original investigators.

## Abbreviations

IBA: International Boxing Association
RSC: Referee-stop contest
RSCI: Referee-stop contest injuries
RSCH: Referee-stop contest head
USA: United States of America

## References

[1] Donnelly RR, Ugbolue UC, Gao Y, Gu Y, Dutheil F, Baker JS. A systematic review and meta-analysis investigating head trauma in boxing. Clin J Sport Med. 2023;33(6):658–74. 37862081 10.1097/JSM.0000000000001195 PMC10597432

[2] Tjønndal A, Haudenhuyse R, de Geus B, Buyse L. Concussions, cuts and cracked bones: a systematic literature review on protective headgear and head injury prevention in olympic boxing. Eur J Sport Sci. 2022;22(3):447–59. 33607924 10.1080/17461391.2021.1872711

[3] Davis P, Waldock R, Connorton A, Driver S, Anderson S. Comparison of amateur boxing before and after the 2013 rules change and the impact on boxers’ safety. Br J Sports Med. 2018;52(11):741–6. 28954796 10.1136/bjsports-2017-097667

[4] deWeber K, Parlee L, Nguyen A, Lenihan MW, Goedecke L. Headguard use in combat sports: position statement of the association of ringside physicians. Physician Sportsmed. 2023:1–10. 10.1080/00913847.2023.2242415 37559553

[5] Alevras AJ, Fuller JT, Mitchell R, Lystad RP. Epidemiology of injuries in amateur boxing: A systematic review and meta-analysis. J Sci Med Sport. 2022;25(12):995–1001. 36195527 10.1016/j.jsams.2022.09.165

[6] Loosemore M. The history and current principles of concussion management in boxing. Aspetar Sport Med J. 2016;5:124–9.

[7] Dickinson P, Rempel P. Prohibiting headgear for safety in amateur boxing? Opinion of the Canadian boxing community: an online poll. Sports medicine-open. 2016;2:1–11. 10.1186/s40798-016-0043-2 PMC4751171 26913220

[8] International Boxing Association. Removal of women’s headguards, Brazil Boxing Confederation gaining provisional membership, and Belgrade being awarded the Women’s World Championships amongst pivotal decisions taken by the IBA Board of Directors: IBA; 2024 [Available from: https://www.iba.sport/news/removal-of-womens-headguards-brazil-boxing-confederation-gaining-provisional-membership-and-belgrade-being-awarded-the-womens-world-championships-amongst-pivotal-decisions-taken-by/

[9] Zazryn T, Cameron P, McCrory P. A prospective cohort study of injury in amateur and professional boxing. Br J Sports Med. 2006;40(8):670–4. 16807306 10.1136/bjsm.2006.025924 PMC2579447

[10] Lota KS, Malliaropoulos N, Blach W, Kamitani T, Ikumi A, Korakakis V, et al. Rotational head acceleration and traumatic brain injury in combat sports: a systematic review. Br Med Bull. 2022;141(1):33–46. 35107134 10.1093/bmb/ldac002 PMC9351374

[11] Loosemore M, Lightfoot J, Beardsley C. Boxing injuries by anatomical location: a systematic review. Med Sportiva: J Romanian Sports Med Soc. 2015;11(3):2583.

[12] Bianco M, Loosemore M, Daniele G, Palmieri V, Faina M, Zeppilli P. Amateur boxing in the last 59 years. Impact of rules changes on the type of verdicts recorded and implications on boxers’ health. Br J Sports Med. 2013;47(7):452–7. 23314931 10.1136/bjsports-2012-091771

[13] Loosemore MP, Butler CF, Khadri A, McDonagh D, Patel VA, Bailes JE. Use of head guards in AIBA boxing tournaments—a cross-sectional observational study. Clin J Sport Med. 2017;27(1):86–8. 27116592 10.1097/JSM.0000000000000322

[14] Paizante RO, Queiroz AC, Fernandes JR, de Brito MA, dos Santos MH, Muñoz EA et al. Headgear increases protection against acute concussion symptoms in amateur olympic boxers. Retos: nuevas tendencias En educación física. Deporte Y Recreación. 2024(60):405–13.

[15] McIntosh AS, Patton DA. Boxing headguard performance in punch machine tests. Br J Sports Med. 2015;49(17):1108–12. 26175022 10.1136/bjsports-2015-095094

[16] Razaghi R, Biglari H, Karimi A. A comparative study on the mechanical performance of the protective headgear materials to minimize the injury to the boxers’ head. Int J Ind Ergon. 2018;66:169–76.

[17] Wolfe EM, Pierrot RG, Slavin BR, Plotsker EL, Samaha GJ, Gishen K, et al. Rolling with the punches: a National electronic injury surveillance system database study of craniofacial injuries in boxing. J Craniofac Surg. 2021;32(4):1576–80. 33741888 10.1097/SCS.0000000000007640

[18] Al-Awady A, Batiste A, Cheng C, Sicard R, Vasan V, Rosenberg J, et al. Nasal injuries in amateur male boxers before and after the 2013 rule change by the international boxing association removing the protective headgear. J Cranio-Maxillofacial Surg. 2025;53(1):6–9. 10.1016/j.jcms.2024.09.005 39490344

[19] AIBA IBA. AIBA World Boxing Championships Doha 2015 - AIBA HeadsUp! Project: IBA. 2015 [Available from: https://www.youtube.com/watch?v=vzscNOFKTuU

[20] International Boxing Association. Karim, Bouzidi AIBAE, Director. #Headsup! will ensure the continuous development of our sport: IBA; 2015 [Available from: https://www.iba.sport/news/karim-bouzidi-aiba-executive-director-headsup-will-ensure-continuos-development-sport/

[21] Davis P, Connorton AJ, Driver S, Anderson S, Waldock R. The activity profile of elite male amateur boxing after the 2013 rule changes. J Strength Conditioning Res. 2018;32(12):3441–6. 10.1519/JSC.0000000000001864 30480653

[22] Da Broi M, Al Awadhi A, Voruz P, Nouri A, Schaller K. The spectrum of acute and chronic consequences of neurotrauma in professional and amateur boxing–A call to action is advocated to better understand and prevent this phenomenon. Brain Spine. 2024;4:102743. 38510617 10.1016/j.bas.2023.102743 PMC10951782

[23] World Medical Association, WMA STATEMENT ON BOXING: WMA. 2023 [Available from: https://www.wma.net/policies-post/wma-statement-on-boxing/

[24] Boxing Canada, RULE CHANGE: BOXING HEADGEAR IS NOW MANDATORY FOR ALL COMPETITORS IN ALL EVENTS IN CANADA: Boxing Canada.; 2019 [Available from: https://boxingcanada.org/annoucements/rule-change-boxing-headgear-is-now-mandatory-for-all-competitors-in-all-events-in-canada/

[25] Hawthorne M. Headgear ban sparks boxing chaos: The Sydney Morning Herald; 2013 [Available from: https://www.smh.com.au/national/headgear-ban-sparks-boxing-chaos-20130629-2p3vl.html

